# Inhibition of MAPK and STAT3-SOCS3 by Sakuranetin Attenuated Chronic Allergic Airway Inflammation in Mice

**DOI:** 10.1155/2019/1356356

**Published:** 2019-09-03

**Authors:** Fernanda P. R. Santana, Rafael C. da Silva, Simone dos S. Grecco, Aruanã J. M. C. R. Pinheiro, Luciana C. Caperuto, Fernanda M. Arantes-Costa, Samuel R. Claudio, Kelly Yoshizaki, Mariângela Macchione, Daniel A. Ribeiro, Iolanda F. L. C. Tibério, Lídio G. Lima-Neto, João H. G. Lago, Carla M. Prado

**Affiliations:** ^1^Department of Biological Science, Federal University of São Paulo, Diadema, SP, Brazil; ^2^Department of Medicine, School of Medicine, University of São Paulo, SP, Brazil; ^3^Department of Bioscience, Federal University of São Paulo, Santos, Brazil; ^4^Universidade Anhanguera de São Paulo, SP, Brazil; ^5^Universidade CEUMA, São Luís, MA, Brazil; ^6^Programa de Pós-Graduação da Rede BIONORTE, Brazil; ^7^Department of Pathology, School of Medicine, University of São Paulo, SP, Brazil; ^8^Center of Natural Sciences and Humanities, Federal University of ABC, Santo André, SP, Brazil

## Abstract

Asthma allergic disease is caused by airway chronic inflammation. Some intracellular signaling pathways, such as MAPK and STAT3-SOCS3, are involved in the control of airway inflammation in asthma. The flavonoid sakuranetin demonstrated an anti-inflammatory effect in different asthma models. Our aim was to clarify how sakuranetin treatment affects MAPK and STAT3-SOCS3 pathways in a murine experimental asthma model. Mice were submitted to an asthma ovalbumin-induction protocol and were treated with vehicle, sakuranetin, or dexamethasone. We assayed the inflammatory profile, mucus production, and serum antibody, STAT3-SOCS3, and MAPK levels in the lungs. Morphological alterations were also evaluated in the liver. LPS-stimulated RAW 264.7 cells were used to evaluate the effects of sakuranetin on nitric oxide (NO) and cytokine production. *In vivo*, sakuranetin treatment reduced serum IgE levels, lung inflammation (eosinophils, neutrophils, and Th2/Th17 cytokines), and respiratory epithelial mucus production in ovalbumin-sensitized animals. Considering possible mechanisms, sakuranetin inhibits the activation of ERK1/2, JNK, p38, and STAT3 in the lungs. No alterations were found in the liver for treated animals. Sakuranetin did not modify *in vitro* cell viability in RAW 264.7 and reduced NO release and gene expression of IL-1*β* and IL-6 induced by LPS in these cells. In conclusion, our data showed that the inhibitory effects of sakuranetin on eosinophilic lung inflammation can be due to the inhibition of Th2 and Th17 cytokines and the inhibition of MAPK and STAT3 pathways, reinforcing the idea that sakuranetin can be considered a relevant candidate for the treatment of inflammatory allergic airway disease.

## 1. Introduction

At least ten percent of the world population is diagnosed with asthma, which is a heterogeneous and complex chronic respiratory disease characterized by Th2 profile cell activation, mast cells, eosinophils, and also neutrophils [1] and involves several mediators/modulators. Moreover, bronchial hyperresponsiveness is associated with an increase in mucus deposition, which obstructs the airflow [1].

The anti-inflammatory effects of sakuranetin, a flavonoid from *Baccharis retusa* DC (Asteraceae), have been demonstrated in different models of lung diseases [2–4]. In this context, we previously had shown that sakuranetin reduces Th2 cytokines (such as IL-5, RANTES, and eotaxin); the number of pulmonary inflammatory cells, mainly eosinophils; IgE levels; and airway, vessel, and lung parenchyma remodeling in a murine model of allergic asthma [5, 6]. The results of herbal-derived natural compounds on eosinophilic infiltration have been previously demonstrated [7–10]. To consider some natural compounds as an alternative treatment to be used in the future is of great interest to the study of the biological mechanisms involved and their possible related toxic effects.

Several signaling pathways have been described to be involved in asthma physiopathology. The MAPK cascade is involved in the activation of immune cells and is responsible for the release of inflammatory mediators [11]. Moreover, the kinases such as p38 and ERK seem to regulate IL-5 and other cytokines [12], while JNK has been described as relevant for IgE class switching [13].

The role of the STAT3-SOCS3 pathway in lung inflammation is full of controversy. SOCS is a family of molecules that suppresses the STAT signaling pathway and regulates Th cell differentiation [14]. The STAT-SOCS relation is poorly studied in asthma, and some studies suggested that this pathway is involved mainly in severe asthma [15, 16]. Some authors have shown that STAT3 inhibition prevents lung inflammation and Th2 cell differentiation in the murine model of asthma [17]. Although SOCS3 is an inhibitor of STAT3, it was demonstrated that the silencing of SOCS3 reduces eosinophil functions in asthmatic patients [18]. Patients classified with severe asthma have high levels of Th17 cells [19], which produce IL-17 cytokines that are not usually inhibited by corticosteroids, the gold-standard treatment for asthma [19]. These patients are classified as uncontrolled asthmatics who exhibit persistent airway eosinophilia/neutrophilia despite continued use of systemic synthetic steroids [20]. It is also important to stress out the relation between the STAT/SOCS pathway and Th17 cells. The induction of severe asthma in SOCS3 knockout mice increased IL-17 levels and also stimulated its differentiation [16]. Moreover, the inhibition of p-STAT3 can be associated with the reduction of IL-17 in an asthmatic mice model [17, 21]. In addition, although these patients represent 10% of the asthmatic population [20, 22], they are responsible for 90% of the total costs of asthma globally [20]. Therefore, it is of utmost importance to find antiasthmatic drug candidates with no or low toxicity to humans. Moreover, considering the importance of Th17, MAPK, and STAT3-SOCS3 in inflammatory responses, inhibition has become an important target for therapeutic strategies in inflammatory diseases.

The current study is aimed at clarifying how Th17, MAPK, and STAT3-SOCS3 are affected by chronic allergic inflammation and if sakuranetin modulates these alterations. We found that sakuranetin reduced eosinophilic inflammation, Th2 and Th17 cytokines, and mucus secretion in airways. The effects of sakuranetin in the inhibition of lung IL-17, STAT3, and MAPK activation seem to explain the mechanics involved in the anti-inflammatory effects of this compound.

## 2. Material and Methods

### 2.1. General Experimental Procedures

Silica gel 60 (Merck Millipore, 230–400 mesh) and silica gel 60 F254 (Merck Millipore) were used, respectively, for column chromatography and analytical TLC (thin-layer chromatography). ^1^H and ^13^C NMR spectra were recorded using CDCl_3_ and CD_3_OD (Sigma-Aldrich) at 300/75 MHz in a Bruker Ultrashield 300 Advance III spectrometer. Chemical shifts are reported in *δ* units (ppm) and coupling constants (*J*) in Hz. LREIMS (70 eV) were measured on a Shimadzu 14B/QP5050A spectrometer.

### 2.2. Plant Material

Aerial parts of *Baccharis retusa* DC. were collected in Campos do Jordão, São Paulo State, Brazil in April 2017. A voucher specimen was prepared and compared with that deposited at the Herbario D. Bento Pickel, Instituto Florestal de São Paulo, São Paulo, Brazil, at number SPSF44897.

### 2.3. Isolation of Sakuranetin from *B. retusa*

Aerial parts of *B. retusa* were dried and milled, and the obtained material (600 g) was extracted with MeOH (8 × 500 mL) at room temperature. After elimination of the solvent under reduced pressure, the crude MeOH extract (50 g) was obtained. This material was resuspended in MeOH : H_2_O (1 : 4) and sequentially partitioned with n-hexane (6 × 250 mL) and CH_2_Cl_2_ (6 × 300 mL). After drying with MgSO_4_ and eliminating the solvents under low pressure, 7 g and 19 g of n-hexane and CH_2_Cl_2_ phases were obtained, respectively. Part of the CH_2_Cl_2_ phase (15 g) was chromatographed over a silica gel column (230–400 mesh; Merck Millipore, Darmstadt, Germany) eluted with n-hexane containing increasing amounts of EtOAc (up to 100%) to afford 68 fractions. After TLC analysis, these fractions were pooled together in seven groups (A-G). Group C (4.2 g) was chromatographed over a silica gel column (230–400 mesh; Merck Millipore) eluted with increasing amounts of EtOAc in CH_2_Cl_2_ (up to 100%) to afford 850 mg of pure sakuranetin.

^1^H NMR (300 MHz, CDCl_3_+CD_3_OD) *δ*H: 7.23 (d, *J* = 8.6 Hz, H-2′/H-6′), 6.81 (d, *J* = 8.6 Hz, H-3′/H-5′), 5.99 (d, *J* = 2.1 Hz, H-6′/H-8′), 5.27 (dd, *J* = 13.0 and 3.0 Hz, H-2), 3.74 (s, OCH_3-7_), 3.04 (dd, *J* = 17.1 and 13.0 Hz, H-3a), and 2.70 (dd, *J* = 17.1 and 3.0 Hz, H-3b). ^13^C NMR (75 MHz, CDCl_3_+CD_3_OD) *δ*C: 196.5 (C-4), 168.0 (C-4′), 163.6 (C-7), 163.0 (C-5), 157.4 (C-8a), 129.0 (C-1′), 127.7 (C-2′/C-6′), 115.3 (C-3′/C-5′), 102.8 (C-4a), 94.9 (C-6), 93.9 (C-8), 79.1 (C-2), 55.3 (OCH_3_), and 42.8 (C-3). LREIMS (70 eV) *m*/*z* (rel. int.): 286 (73), 193 (29), 180 (39), 167 (100), 120 (44), 107 (12), and 95 (22).

### 2.4. Animal Preparation and Experimental OVA Protocol

All animal and cell experiments performed in this study were carried out according to protocols approved by the Animal Care Committee of the School of Medicine, University of São Paulo, Brazil (CEUA-FMUSP, number 105/15) and of the Federal University of São Paulo (CEUA-UNIFESP, number 8319281014). All animals received humane care in compliance with the Principles of Laboratory Animal Care formulated by the National Society for Medical Research and the Guide for the Care and Use of Laboratory Animals prepared by the National Academy of Sciences, USA. Moreover, the animal studies are reported in accordance with the ARRIVE guidelines [23].

Male BALB/c mice aged 6-8 weeks (20-25 g) obtained from the Central Animal Care Facility at the University of São Paulo were housed in groups of 5 per individually ventilated cages with a 12-hour light/dark cycle and free access to food and water. In order to induce airway allergic inflammation, animals were assigned randomly and divided into four experimental groups: (a) SAL (submitted to saline protocol and treated with vehicle), (b) OVA (submitted to the ovalbumin sensitization and treated with vehicle), (c) SK (submitted to the OVA sensitization and treated with sakuranetin), and (d) DX (submitted to OVA sensitization and treated with dexamethasone). Ovalbumin (OVA) (50 *μ*g i.p.) (grade IV, Sigma-Aldrich, St. Louis, MO, USA) in the presence of 6 mg of an Al(OH)_3_ adjuvant (Pepsamar, Sandei-Synthelabo SA, RJ, Brazil) diluted in a 0.2 mL saline solution was used to immunize animals as previously described on days 1 and 14 [6]. One week after the second immunization, mice were placed in a Plexiglas box (30 cm × 15 cm × 20 cm) coupled to an ultrasonic nebulizer (US-1000; ICEL, SP, Brazil) and exposed to 1% OVA aerosol for 30 min on days 22, 24, 26, and 28. All OVA-sensitized animals received 20 mg/kg^–1^ daily (10 *μ*L, intranasal) of sakuranetin diluted in DMSO (Sigma-Aldrich) and saline 0.9% (1 : 4), since day 22 until euthanasia [6]. Another group of animals used as a positive control was sensitized with OVA and treated with dexamethasone by i.p. injection (5 mg/kg^–1^) at the same time points of sakuranetin treatment [6]. Control mice (SAL group) received the adjuvant (i.p.) and were exposed, at the same time points, to a saline aerosol (0.9% NaCl). The SAL and OVA groups were treated with vehicle (1 : 4 of DMSO and saline 0.9%). The lung, peripheral blood, and inner bone narrow liquid were collected on day 29, thirty minutes after the last treatment dose of sakuranetin, dexamethasone, or vehicle.

### 2.5. Inflammatory Cell Counting

After anesthesia (50 mg/kg of thiopental, i.p.), peripheral blood was collected and used to count the numbers of leucocytes in the Neubauer chamber. The different cells were analyzed in the sanguine smear that was stained using Diff-Quick (Instant-Prov, Newprov, Pinhais, PR, Brazil), and a total of 100 cells were counted according to their morphological pattern. Laparotomy was performed, and blood was collected by venipuncture in the vena cava for serum antibody measurement. Mice were euthanized by exsanguination, and bronchoalveolar lavage fluid (BALF) samples were collected after the infusion of 0.5 mL of sterile saline in the lungs, repeated for three times as previously described [6]. BALF was centrifuged at 1000 × *g* for 10 min at 4°C, and the cell pellet was suspended in 300 *μ*L of saline. The total number of viable cells was determined in a Neubauer chamber (Carl Roth, Karlsruhe, Germany). Cytocentrifuge slides of BALF samples were stained with Diff-Quick (Instant-Prov, Newprov, Pinhais, PR, Brazil) to count differential cells (at least 300) according to their morphological pattern (eosinophils, neutrophils, macrophages, and lymphocytes) in an optical microscope [6].

### 2.6. Histological Analyses

Lungs were removed *en bloc*. The left lung was quickly frozen in liquid nitrogen and stored at -80°C to ELISA and Western blotting analysis. The right lung was fixed and embedded in paraffin. The nose was removed and decalcified in 5% EDTA for 14 days [24]. Three- to five-micrometer-thick sections of paraffin-embedded nose and lung tissues were stained with Periodic Acid-Schiff/Alcian Blue (PAS-AB) as previously described [24]. The morphometric analyses were performed using an integrating eyepiece with a coherent system consisting of a grid with 100 points and 50 lines of known length coupled to a conventional light microscope [25] (Nikon E200, Tokyo, Japan). We counted how many intersecting crosses were there with the basement membrane to determine the basement membrane perimeter. Because the perimeter of the basement membrane was similar among the experimental groups, we used the points coincident with the bronchial epithelia to determine the area (10^4^*μ*m^2^) in three to five airways per animal. To determine the mucus area, we evaluated ten to fifteen random noncoincident microscopic fields and measured the acid and neutral mucus in bronchial and nasal epithelia by counting the points incident to acid or neutral mucus and the total points in epithelia.

Thymus, liver, and spleen were collected, weighed, and quickly frozen in nitrogen and stored at -80°C for further analysis. The liver was stained with H&E, and its morphometry was evaluated by conventional light microscopy in order to detect any signals of toxicity. All histological analyses were performed by an observer unaware of the experimental protocol. For this purpose, the following parameters were considered: the presence of inflammatory infiltrate, necrosis, congested vessels, and malignant transformation. To determine if cytotoxicity was present, the following metanuclear changes were also evaluated: pyknosis, karyorrhexis, and karyolysis. A total of 1000 hepatocytes per animal were evaluated.

### 2.7. Antibody Measurements

Approximately 0.3 mL of blood was collected, diluted into 600 *μ*L of saline solution, and centrifuged at 3,000 rpm for 10 min at 4°C, in order to obtain the serum. We then added IgE-, IgG1-, or IgG2a-specific biotinylated antibodies (SouthernBiotech, Birmingham, AL, USA) into a microplaque covered with OVA antigen and incubated with serum/sera samples. The developing solution containing the enzymatic conjugate of streptavidin-peroxidase, substrate, and chromogen was added, and the color reaction was read in a 490 nm spectrophotometer; it was found to be proportional to the quantity of the immunoglobulins in the sample. The results were expressed as mean absorbance ± standard error of the serial dilutions of the samples of each group [26].

### 2.8. Lung Homogenate Preparation

Using a Polytron PTA 20S (Brinkmann Instruments Model PT 10/35), the lung homogenate was lysed on ice with the aid of 1.5 mL of buffer (1% g/ml SDS, 100 mM Trisma (pH 7.5), 10 mM EDTA, 100 mM tetrasodium pyrophosphate dihydrate, 100 mM sodium fluoride, 10 mM sodium orthovanadate). The total protein concentration was measured by the Bradford method (Bio-Rad, Hercules, CA, USA).

### 2.9. Cytokine Measurements

The levels of IL-4, IL-13, IL-17, RANTES, TNF-*α*, and IFN-*γ* in the lung homogenate were assessed by an ELISA kit according to the manufacturer's instructions (R&D Systems, Minneapolis, MN, USA). The cytokines analyzed were obtained using standard curves, and each sample was assayed in duplicates and read at 450 nm.

### 2.10. Lung Western Blotting Analysis

Western blot was performed using the protocol modified from Caperuto et al. in 2008 [27]. 50 *μ*g of protein was loaded on an SDS-PAGE gel (10% bis-acrylamide) and then electrotransferred from the gel to a nitrocellulose membrane (10 minutes at 25 V constant) in a semidry transfer (Trans-Blot Turbo, Bio-Rad). Nonspecific binding protein in the nitrocellulose membrane was blocked with 5% milk/TBST and then incubated with anti-phospho-p38 MAPK (1 : 2,000, mouse IgG1, 9216), anti-p38 MAPK (1 : 1,000, rabbit IgG, 8690), anti-phospho-p44/p42 MAPK (ERK1/2) (1 : 2,000, rabbit IgG, 4370), anti-p44/p42 MAPK (1 : 2,000, mouse IgG1, 4696), anti-phospho-SAPK/JNK (1 : 2,000, mouse IgG1, 9255), anti-SAPK/JNK (1 : 1,000, rabbit, 9252), anti-phospho-STAT-3 (1 : 1,000, rabbit, 9131), anti-STAT-3 (1 : 1,000, mouse IgG2a, 9139), anti-SOCS-3 (1 : 1,000, rabbit, 2923) (Cell Signaling Technology, Danvers, MA, USA), and anti-*β*-actin (1 : 5000, mouse, A5316) (Sigma-Aldrich) diluted in blocking buffer for 4°C overnight. Bound antibodies were detected with horseradish peroxidase-conjugated (HRP-conjugated) anti-mouse IgG or anti-rabbit IgG (1 : 10,000, 7076 and 7074) (Cell Signaling Technology, Danvers, MA, USA) and visualized using UVItec (UVITEC Cambridge, England, United Kingdom) by chemiluminescence. The image program UVIband (UVITEC Cambridge) was used to quantify the intensities of the bands. The expression of all proteins was normalized to the constitutive *β*-actin protein, and all phosphorylated protein expression was normalized to its specific total protein. All the results were expressed as percentage related to the control (control group).

### 2.11. Culture and Treatment of RAW 264.7 Cell

The RAW 264.7 cell line was previously obtained from the American Collection of Cell Culture (ATCC, USA). The cells were cultured in appropriate bottles and sterilized with RPMI-1640 medium (Sigma-Aldrich) supplemented with 1% antibiotic+amphiphilic solution (Sigma-Aldrich) and 10% fetal bovine serum (FBS, Sigma-Aldrich) incubated at 37°C in a humidified atmosphere containing 5% CO_2_ and 95% atmospheric air.

The cell viability assay was evaluated by MTT methods. The RAW 267.4 cells (2 × 10^5^ cells in 200 *μ*L of RPMI medium+2% FBS per well in a 96-well plate) were treated with sakuranetin at 5, 20, and 50 *μ*g/mL concentrations. After 48 hours, the supernatant was removed and 100 *μ*L of the medium was added with 0.5% MTT (3-(4,5-dimethylthiazol-2-yl)-2,5-diphenyltetrazolium bromide) (Sigma-Aldrich) to form crystals of formazan in viable cells. The plate was incubated for 4 hours, and 100 *μ*L of DMSO (Sigma-Aldrich) was added and maintained at room temperature to complete the solubilization of formazan crystals. The reading was performed immediately in a spectrophotometer at a wavelength of 570 nm.

The production of nitric oxide (NO) was determined by measuring nitrite as previously described [28]. The cells were stimulated by lipopolysaccharide (LPS) (30,000 UE/10 *μ*g/100 *μ*L/well) obtained from *Escherichia coli* (Sigma-Aldrich) and treated with sakuranetin at 5, 20, and 50 *μ*g/mL concentrations for 48 hours. For analysis of NO, 100 *μ*L of cell supernatants were incubated with 100 *μ*L of a Griess reagent (Sigma-Aldrich), and the absorbance was determined at 540 nm. A NO_2_ standard curve (0-300 *μ*M) was used to compare the evaluation. The NO_2_ results are expressed as *μ*M.

Total RNA was extracted using a RNeasy Mini Kit (Qiagen, Hilden, Germany) according to the manufacturer's instructions in a QIAcube automatic DNA extractor. Afterwards, the total RNA was treated with DNase (Qiagen) and then reverse transcribed using 200 U of SuperScript II Reverse Transcriptase (Thermo Fisher Scientific, Waltham, Massachusetts, USA) to obtain cDNA. The qPCR assays were carried out in 96-well plates using a double-stranded DNA dye (GoTaq® real-time PCR Master Mix from Promega) for the IL-6 (primer forward: CAGGCTCCGAGATGAACAAC; primer reverse: GGTGGAGAGCTTTCAGCTCATAT) and IL-1*β* (primer forward: GGCAGCTACCTGTGTCTTCC; primer reverse: ATATGGGTCCGACAGCACGAG) mRNA expression in a QuantStudio™ 6 Flex Real-Time PCR System as previously described. GAPDH mRNA (primer forward: TGAAGGTCGGTGTGAACGG; primer reverse: CGTGAGTGGAGTCATACTGGAA) was used as an endogenous reference gene. mRNA relative expression was calculated using the 2^−ΔCt^ method [28]. Results represent the mean values obtained from two independent experiments, with assays performed in triplicate.

### 2.12. Data and Statistical Analysis

Statistical analysis was performed using GraphPad Prism 7 software (GraphPad Software Inc., San Diego, CA, USA). The parametric data were analyzed by one-way ANOVA, followed by Holm-Sidak's method. All data were expressed as mean ± SEM, and the statistical significance level was adjusted to 5% (*p* < 0.05).

## 3. Results

### 3.1. Chemical Characterization of Sakuranetin

**Figure 1:**
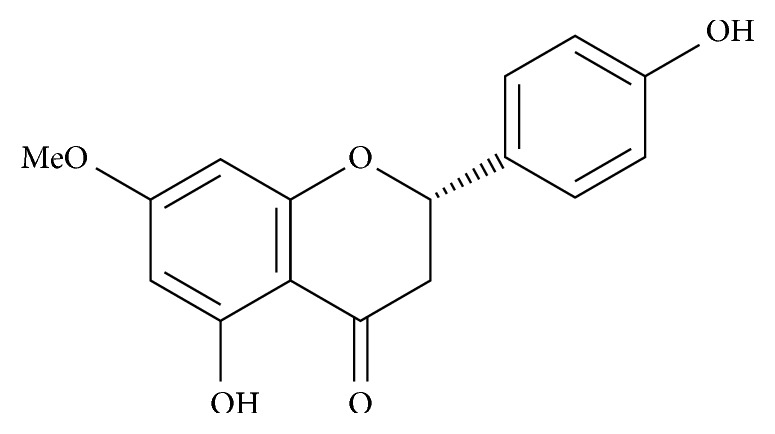
Sakuranetin structure, 5,4′-dihydroxy-7-methoxy flavanone, isolated from *B. retusa*.

The ^1^H NMR spectrum displayed characteristic double doublets attributed to H-2, H-3a, and H-3b of flavanones [22] at *δ* 5.27 (*J* = 13.0 and 3.0 Hz), 3.04 (*J* = 17.1 and 13.0 Hz), and 2.70 (*J* = 17.1 and 3.0 Hz), respectively. Additionally, this spectrum showed peaks of aromatic hydrogens at *δ* 6.81 (d, *J* = 8.6 Hz, H-3′/H-5′), 7.23 (d, *J* = 8.6 Hz, H-2′/H-6′), and 5.99 (d, *J* = 2.1 Hz, H-6′/H-8′) as well as one singlet attributed to the methoxyl group at *δ* 3.74. The ^13^C NMR (nuclear magnetic resonance) spectrum showed peaks assigned to aromatic carbons C-4a to C8a and C-1′ to C-6′ at a range of *δ* 94–168, to carbonyl C-4 at *δ* 196.5, to oximethine carbon C-2 at *δ* 79.1, to methylene carbon C-3 at *δ* 42.8, and to the methoxyl group at *δ* 55.3. These data, associated to fragmentation observed in the LREIMS (low resolution electron ionization mass spectrometry), are consistent with the structure of 5,4′-dihydroxy-7-methoxy flavanone (sakuranetin), in concordance with data previously reported in the literature [29] (Figure 1).

### 3.2. Sakuranetin Treatment Attenuated the Immune and Inflammatory Responses

The total cells from bone marrow and the total and differential cells from the peripheral blood and in BALF, as well as the levels of IgE, IgG1, and IgG2a are shown in Table 1. Neither OVA protocol nor sakuranetin nor dexamethasone treatments have modified the production of bone marrow total cells. However, following sensitization and challenges, the number of circulating eosinophils (*p* < 0.05) as well as total cells, macrophages (*p* < 0.001 for both), lymphocytes (*p* < 0.05), neutrophils (*p* < 0.05), and eosinophils (*p* < 0.001) in BALF were increased compared to the control SAL. Both sakuranetin and dexamethasone treatments reduced the circulating eosinophils (*p* < 0.05), macrophages (*p* < 0.001), and eosinophils (*p* < 0.001) in BALF. Dexamethasone treatment also reduced the number of lymphocytes in BALF (*p* < 0.05) compared to the OVA group.

To analyze whether the OVA protocol was able to stimulate an anaphylactic inflammatory response similar to human asthma, we measured the levels of IgE, IgG1, and IgG2a by ELISA assay. The OVA group showed increased levels of IgE and IgG2a (*p* < 0.05 for both) compared with SAL. The sakuranetin treatment reduced IgE levels (*p* < 0.05) in serum, different from that of the dexamethasone treatment. No effects of sakuranetin and dexamethasone were observed in the serum levels of IgG2a. No alterations were observed in the IgG1 levels.

Because immune responses can induce thymus and spleen hypertrophy, we evaluated the mass percentage of these organs in relation to total body weight. We found that OVA sensitization increased the thymus (*p* < 0.05) and spleen (*p* < 0.01) percentage compared to SAL. Interestingly, both treatments attenuated the lung inflammation and induced a reduction in the thymus (*p* < 0.01 to SK and *p* < 0.001 to DX) and spleen mass (*p* < 0.05 to SK and *p* < 0.001 to DX) compared to OVA (Table 1).

### 3.3. Sakuranetin Treatment Avoids the Increase of Bronchial Epithelium Area and Acid Mucus Production in Bronchial and Nasal Epithelia

**Figure 2:**
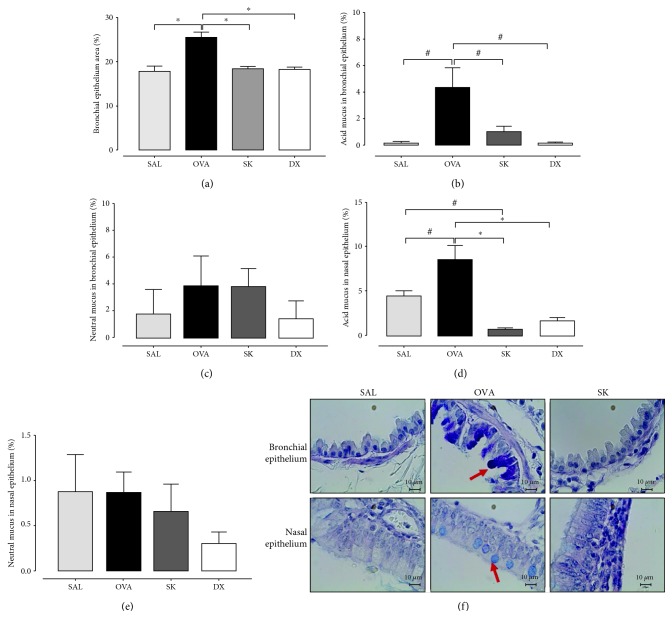
Bronchial epithelium area and acid mucus production were reduced by the sakuranetin and dexamethasone treatments compared with the OVA group: (a) bronchial epithelium area; (b) acid mucus in bronchial epithelia; (c) neutral mucus in bronchial epithelia; (d) acid mucus in nasal epithelia; (e) neutral mucus in nasal epithelia. (f) Images represent lung and nasal septal slices stained with PAS+AB from the SAL, OVA, SK, and DX groups at a magnification of ×20. ^∗^*p* < 0.001 and ^#^*p* < 0.05. Data represent mean ± SEM from 5 to 8 mice per group.

Considering that allergic rhinitis is strongly related to asthma and mucus production is an important feature of asthmatic patients, we stained lung and nasal slices with PAS+AB. The bronchial epithelium area was increased in the OVA group compared with the SAL group (*p* < 0.001) (Figure 2(a)). Both sakuranetin and dexamethasone treatments reduced the bronchial epithelium area (*p* < 0.001). Acid mucus was increased in bronchial (Figure 2(b)) and nasal epithelia (Figure 2(d)) in OVA-sensitized animals compared with the SAL group (*p* < 0.05). Both sakuranetin and dexamethasone treatments reduced the acid mucus production (*p* < 0.05 and *p* < 0.001, respectively). There was no significant difference in neutral mucus production among the experimental groups in both bronchial and nasal epithelia (Figures 2(c) and 2(e)).  Figure 2(f) demonstrates the representative lung and nasal slices from the saline, OVA, and SK groups. The blue stain indicates acid mucus in epithelia (arrows).

### 3.4. Sakuranetin Treatment Reduced Th2 and Th17 Cytokine Levels in Lung Homogenate

**Figure 3:**
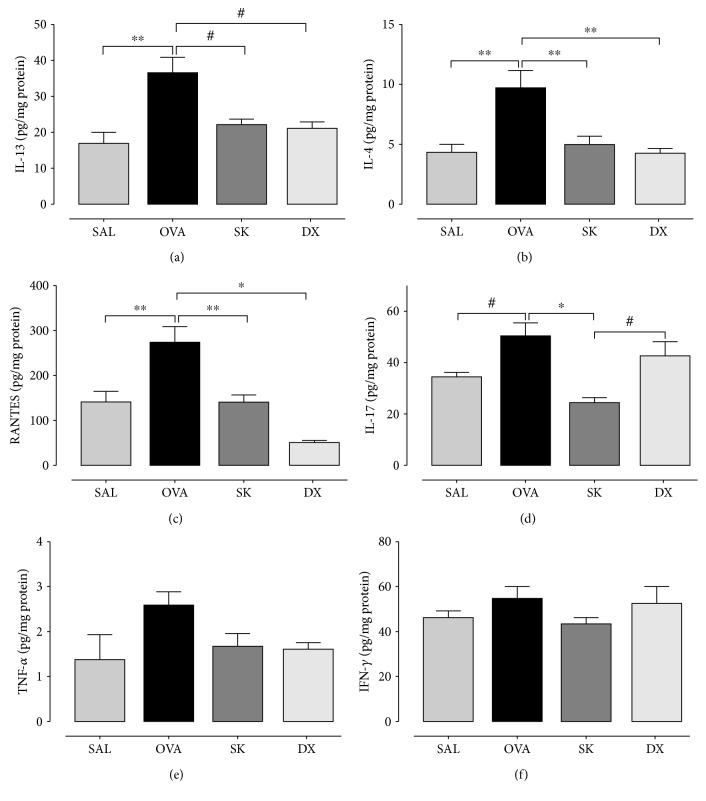
Sakuranetin inhibits Th2 and Th17 cytokines in lung content (pg/mg) that was increased in the OVA group: (a) IL-13; (b) IL-4; (c) RANTES; (d) IL-17; (e) TNF-*α*; (f) IFN-*γ* measured by ELISA technique in lung homogenate. ^∗^*p* < 0.001, ^∗∗^*p* < 0.01, and ^#^*p* < 0.05. Data represents mean ± SEM from 6 to 9 mice per group.

The balance among Th1, Th2, and Th17 is important to the inflammatory responses in asthma. To investigate whether sakuranetin treatment controls the secretion of cytokines, we measured the levels of IL-13 (Figure 3(a)), IL-4 (Figure 3(b)), RANTES (Figure 3(c)), IL-17 (Figure 3(d)), TNF-*α* (Figure 3(e)), and IFN-*γ* (Figure 3(f)) in lung homogenate by ELISA. The OVA group showed higher levels of IL-13, IL-4, RANTES (*p* < 0.01), and IL-17 (*p* < 0.05) compared with the SAL group. Mice treated with sakuranetin and dexamethasone showed a decrease of IL-13 (*p* < 0.05), IL-4 (*p* < 0.01), and RANTES (*p* < 0.01) levels compared with the OVA group. Interestingly, the level of IL-17 was attenuated only by sakuranetin treatment in OVA-sensitized animals compared with the OVA and DX groups (*p* < 0.001 and *p* < 0.05, respectively). In this model, we did not observe an alteration in the levels of TNF-*α* and IFN-*γ* among the groups.

### 3.5. Sakuranetin Attenuated the Activation of MAPK Pathway on Lung

**Figure 4:**
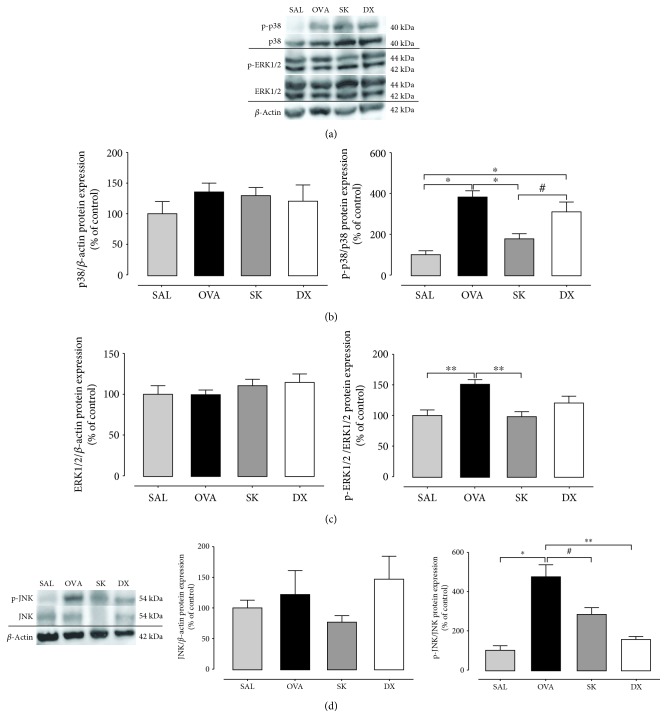
Sakuranetin inhibits the activation of the MAPK pathway. (a) Image intensity of p38, p-p38, ERK1/2, and p-ERK1/2. (b) Expression of p38 and p-p38. (c) Expression of ERK1/2 and p-ERK1/2. (d) Expression and image intensity of JNK and p-JNK in lung tissue measured by immunoblotting. Sakuranetin reduced the phosphorylation of p38, ERK1/2, and JNK that was enhanced in the asthmatic group. ^∗^*p* < 0.001, ^∗∗^*p* < 0.01, and ^#^*p* < 0.05. Data represent mean ± SEM from 5 to 8 mice per group.

MAPK is an important pathway involved in lung inflammation and also in mucin production [13]. We had shown that OVA sensitization activated the MAPK pathway (p-38 (*p* < 0.001), p-ERK1/2 (*p* < 0.01), and p-JNK (*p* < 0.001); Figures 4(a)–4(c)) compared with the SAL group. Sakuranetin inhibited the phosphorylation of p-38, ERK1/2, and JNK (*p* < 0.001, *p* < 0.01, and *p* < 0.05, respectively), whereas dexamethasone attenuated only the phosphorylation of the JNK protein (*p* < 0.01) when compared with the OVA group.

### 3.6. Sakuranetin Attenuated the Activation of STAT3-SOCS3 Pathway on Lung

**Figure 5:**
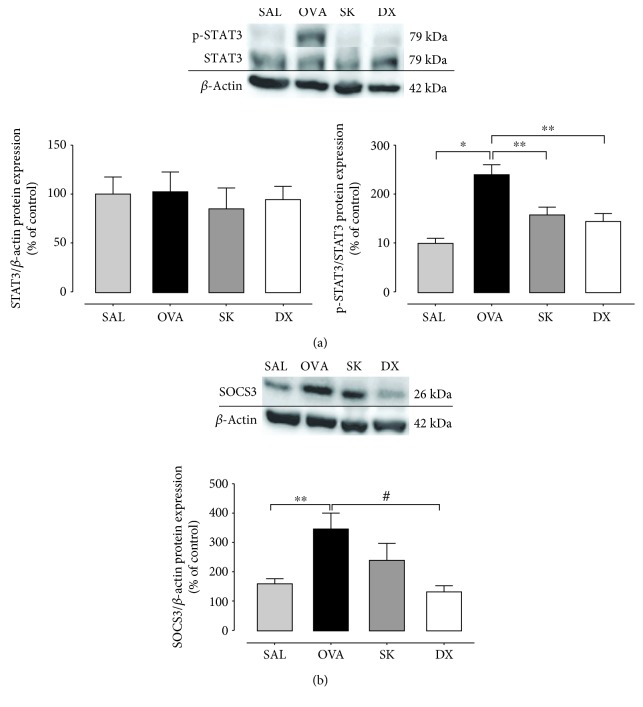
Sakuranetin inhibits the activation of the STAT3-SOCS3 pathway. Expression and intensity of (a) STAT3 and p-STAT3 and (b) SOCS3 in lung tissue detected by immunoblotting. Sakuranetin reduced the phosphorylation of STAT3 that was enhanced in the asthmatic group. ^∗^*p* < 0.001, ^∗∗^*p* < 0.01, and ^#^*p* < 0.05. Data represent mean ± SEM from 5 to 8 mice per group.

The role of STAT3-SOCS3 in asthma is still controversial. The OVA sensitization induced an activation of STAT3 (*p* < 0.001) (Figure 5(a)) and increased levels of SOCS3 (*p* < 0.01) (Figure 5(b)) in the lungs compared with the SAL group. Both treatments, sakuranetin and dexamethasone (*p* < 0.01 for both), inhibited the activation of STAT3 in sensitized animals. However, only dexamethasone attenuated the levels of SOCS3 in the lungs (*p* < 0.05) compared with the OVA group.

### 3.7. Sakuranetin Did Not Induce *In Vivo* Toxicity or Affect Cell Viability *In Vitro*

**Figure 6:**
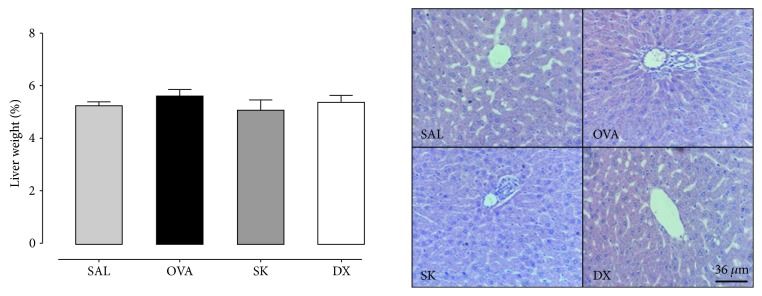
Sakuranetin did not induce liver morphological alterations in vivo. Liver percentage per animal weight and the histopathological slices of liver tissues. The liver morphological examinations were performed with H&E staining at a magnification of ×400; no morphological differences were observed among the groups. Data represent mean ± SEM from 7 to 8 mice per group.

**Figure 7:**
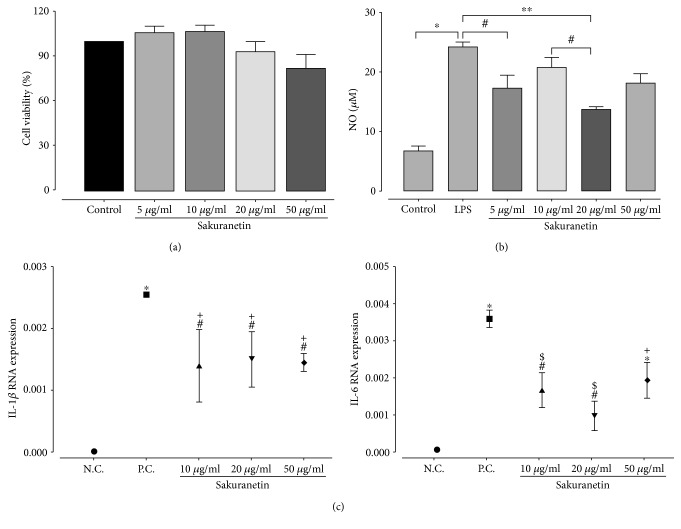
Sakuranetin did not affect cell viability *in vitro* and reduced NO, IL-1*β*, and IL-6 levels in the LPS-stimulated RAW 264.7 cell line. (a) Cell viability with different doses of sakuranetin. (b) NO measurement after LPS stimulation with pretreatment of sakuranetin doses. ^∗^*p* < 0.001, ^∗∗^*p* < 0.01, and ^#^*p* < 0.05. All doses of SK were different compared to the control group (*p* < 0.05). (c) IL-1*β* and IL-6 RNA expression after LPS stimulation with pretreatment of sakuranetin doses; ^#^*p* < 0.05 compared with SAL, ^∗^*p* < 0.001 compared with SAL, ^+^*p* < 0.05 compared with OVA, and ^$^*p* < 0.001 compared with OVA and SK 20 *μ*g/mL. Data represent mean ± SEM from a pull of cells performed in duplicate.

In order to evaluate whether sakuranetin induced liver toxicity, we measured the liver mass and performed histopathology. All animals from the control group did not present any histopathological changes, such as the presence of inflammatory infiltrate, necrosis, congested vessels, or malignant transformation. The treatment with sakuranetin did not induce any remarkable changes to the experimental group (Figure 6). In the same way, sakuranetin did not induce cytotoxicity in liver cells as depicted by pyknosis, karyorrhexis, and karyolysis frequencies (Table 2). Moreover, sakuranetin did not induce *in vitro* cell toxicity and reduced NO and cytokines in cultured RAW 264.7 macrophages (Figures 7(a) and 7(b)).

### 3.8. Sakuranetin Reduced NO, IL-1*β*, and IL-6 RNA Expression in RAW 264.7 Cell Line

We used cultures of RAW 264.7 in order to evaluate the *in vitro* effects of sakuranetin. We found that in doses from 5 to 50 *μ*g/mL, sakuranetin did not reduce the cell viability. RAW 264.7 macrophages were stimulated with LPS in order to induce the release of NO. Sakuranetin reduced the release of NO induced by LPS stimulation in macrophages mainly in doses of 5 and 20 *μ*g/mL. Considering the release of proinflammatory cytokines in RAW 264.7 cells, IL-1*β* and IL-6 mRNA levels induced by LPS were downregulated by sakuranetin treatment at all doses (Figure 7(c)).

## 4. Discussion

In the current study, we showed that the activation of MAPK and STAT3-SOCS3 pathway, associated with lung levels of IL-17, are induced by ovalbumin in mice. Moreover, our data indicates that sakuranetin treatment reduces eosinophils in peripheral blood and lungs, mucus production, and IL-13 levels, important characteristics of asthmatic patients. These antiasthmatic effects can be mediated by the inhibition of IL-17, MAPK, and STAT3 in the lungs. These mechanics are new and have clinical relevance considering the anti-inflammatory effects of this potential candidate compound. Finally, we showed that sakuranetin did not induce toxic effects in the liver of the treated animals and *in vitro* in cell viability.

Sakuranetin is a natural flavonoid with anti-inflammatory and antioxidant potentials [5, 6]. In this context, it has been demonstrated that sakuranetin is able to inhibit lung inflammation in different models including acute lung injury induced by LPS [2] and lung emphysema induced by elastase [4]. Our previous studies also demonstrated the effects of sakuranetin in a murine model of asthma which includes the reduction of the eosinophil airway inflammation, bronchial hyperresponsiveness, and airway remodeling. These data also indicate that sakuranetin has similar effects to those obtained with dexamethasone, a glucocorticoid [5, 6].

Inflammatory changes characterized by an increase of OVA-specific IgE levels, eosinophils in peripheral blood and in the lungs, and increased levels of RANTES, IL-13, IL-4, and IL-17 cytokines, without alterations in Th1 cytokines were observed in sensitized animals. These inflammatory and allergic responses are ameliorated with sakuranetin treatment as well as with dexamethasone, supporting previous data from our group and others [5, 6, 30–32]. Increased levels of IL-4 are associated with IgE synthesis mediated by a switching of activated B lymphocytes which promotes CD4+ Th2 cell activation [33]. Moreover, the inhibition of IL-4 reduces IgE production [34].

Sakuranetin suppressed IL-4 and IgE production, suggesting that this compound inhibits Th2 responses in this model. There is no alteration of Th1 cytokines in OVA-sensitized animals which is consistent with our previous results [6], although IgG2a levels, which are associated to Th1 responses [35], were increased in these animals. The dexamethasone treatment did not reduce the levels of IgE and IgG2a, suggesting that the sakuranetin treatment was more effective for the sensitization compared to DX. Similar data was demonstrated by Jang et al. in 2018, who showed that DX treatment did not reduce the levels of the OVA-specific IgE antibody [36]. Sakuranetin treatment also reduced the thymus and spleen hypertrophy, suggesting that it suppresses the immune response.

Because severe asthmatic patients did not respond adequately to corticosteroids, it is of paramount importance to find anti-inflammatory drugs targeting several asthmatic factors, with minimal or no side effects. The purpose of the present study was to establish the mechanisms involved in the effects of sakuranetin. In this context, it is important to stress out the ability of sakuranetin to reduce IL-17 levels in this OVA-sensitized animal model, a capacity that was not observed in dexamethasone treatment. This data is relevant because IL-17 is associated to the severe asthma profile [19, 37] which is known to be resistant to corticosteroid treatment.

The sakuranetin effects on MAPK pathway activation was evaluated [38], since MAPK is known as a strong pathway involved in asthmatic inflammatory activation [39]. The OVA-induced protocol induces the activation of JNK, ERK1/2, and p38 proteins in the lungs. Our findings are consistent with several studies that prove the upregulation of MAPK proteins in the asthma model [11, 39]. MAPK fosters T cell differentiation, and its inhibition has been tested in preclinical studies in corticosteroid-resistant patients [40]. Pharmacological inhibition, or ERK1/2 knockout mice, blocks asthmatic responses in an asthma model [13, 41]. The activation and expression of JNK and p38 increased the production of RANTES in bronchial epithelium cells [42]. Sakuranetin inhibits the MAPK protein phosphorylation evaluated in this study, which was different from that of corticosteroids which diminished only JNK levels.

Another important pathway involved in the modulation of inflammation is the STAT3-SOCS3. However, its role in asthma is not completely understood. In the present study, there were increased levels of SOCS3 and phosphorylated STAT3 (p-STAT3) in OVA-sensitized animals. Moreover, both sakuranetin and dexamethasone treatments reduced the phosphorylation of STAT3. Gavino et al. in 2016 showed that STAT3 inhibition reduced the production of IL-17 [17]. In this context, the effects of sakuranetin reducing the levels of IL-17 can be associated to the inhibition of p-STAT3. The augmenting of SOCS3 expression in T cells has been associated with asthma severity [43]. However, SOCS3 induces a negative feedback in the regulation of STAT3 [14]. Interestingly, sakuranetin maintained the levels of SOCS3 and reduced IL-17 levels in the lungs, whereas dexamethasone treatment reduced the levels of SOCS3 but maintained high levels of IL-17 in the lungs. Sun et al. in 2018 showed that severe asthmatic animals have high levels of SOCS3 protein, and this anti-inflammatory protein depletion in mice induces an increase of IL-17. The stable increase of SOCS3 levels in sakuranetin-treated animals may strengthen the inhibition of STAT3 phosphorylation and reduction of IL-17 levels [21]. These results are important since STAT3 is required for Th17 lymphocyte differentiation and for the release of cytokines; it has also been associated with severe forms of asthma and the development of persistent airway inflammation [17, 21]. However, further specific studies need to be performed to clarify the role of STAT3-SOCS3 in asthma.

Considering the effects of sakuranetin in lung inflammation, it is plausible that the effects can also depend on the NF-*κ*B inhibition, since both MAPK and STAT3 pathways can activate NF-*κ*B in the end [44]. This idea is reinforced by our previous studies that showed NF-*κ*B inhibition by sakuranetin treatment in a model of asthma [6] and lung emphysema [4] as well as in acute lung injury [2].

One of the important features of asthma is the mucus deposition and alterations in bronchial epithelia. Moreover, mucus production usually affects nasal epithelia and it is known that rhinitis is strongly associated to the development and the worsening of asthma symptoms [45]. OVA inhalation induces mucus deposition in airway epithelial cells, which is positively correlated with the reduction in lung function [46]. We showed that in this model, OVA also increased acid mucus in both bronchial and nasal epithelia. Curiously, sakuranetin inhibits the acid mucus deposition in airway and nasal epithelia. This could be pertinent for the treatment of asthmatic patients.

The increase of IL-13 and IL-4 levels was related with the presence of mucus in airway asthmatic cells. However, the MAPK pathway can be responsible for the IL-13 increase, which will stimulate goblet cells and consequently the mucus production [47].

Considering the potential of sakuranetin in asthma control, it is relevant to know whether this compound induces possible side effects. The flavanone bioavailability seems to be dependent on their hepatic metabolic stability [48, 49]. Therefore, we evaluated the liver morphometry and no signal of liver injury in treated animals was found.

To complement our studies, we also test the effects of sakuranetin *in vitro* in the RAW 264.7 macrophage cell line. It is interesting that in doses of 5 to 50 *μ*g/mL, sakuranetin did not affect cell viability. Because sakuranetin is a flavonoid with antioxidant properties, we used macrophages pretreated with sakuranetin, and we found that sakuranetin inhibits the release of LPS-induced NO in the cells. Proinflammatory mediators released by macrophages are considered as immune accelerators that drive macrophage activation [28]. The gene expression of proinflammatory cytokines such as IL-6 and IL-1*β* in LPS-stimulated RAW 264.7 cells were inhibited by sakuranetin treatment. Although the mechanisms of LPS-induced cytokine and NO release in RAW 264.7 cells are very different from the OVA-induced allergic inflammation in vivo, we choose this model to induce a known inflammatory response *in vitro* and show the isolated effects of sakuranetin inhibiting proinflammatory cytokines. Collectively, these data suggest that sakuranetin has an important anti-inflammatory effect *in vivo* and *in vivo*.

## 5. Conclusion

In conclusion, our study demonstrated that IL-17 was increased and MAPK and STAT3-SOCS3 are activated in the lungs of sensitized animals. Sakuranetin inhibits the release of proinflammatory cytokines and NO in RAW 264.7 *in vitro* without induced cellular death. The intranasal sakuranetin in an *in vivo* murine model of allergic asthma was able to inhibit the production of IgE antibodies, eosinophilic recruitment to the lung, and mucus production in bronchial and nasal epithelia. These effects can be associated to a reduction in both Th2 and Th17 cytokines. Our findings suggest that MAPK and STAT3 pathways are molecular mechanisms that can explain the antiallergic and anti-inflammatory effects of sakuranetin. This study reinforces the idea that sakuranetin can be considered a relevant candidate to the treatment of inflammatory allergic airway disease and, at least in mice and in macrophage cells, sakuranetin did not induce toxic effects.

## Figures and Tables

**Table 1:** Inflammatory and immune effects of sakuranetin treatment. OVA exposure increased inflammatory cells in blood (cells/mm^3^), bronchial lavage fluid (BALF 10^4^ cell/mL), IgE, IgG1, IgG2a in serum (OD), and the mass of immune organs (%). ^##^*p* < 0.05 and ^#^*p* < 0.001 compared with SAL; ^∗∗^*p* < 0.05 and ^∗^*p* < 0.001 compared with OVA; ^€^*p* < 0.001 compared with SK. Data represent mean ± SEM from 8 to 11 mice per group.

Data/groups	SAL	OVA	SK	DX
Bone marrow × 10^6^ cells/mL				
Total cells	38.25 ± 6.90	50.64 ± 8.05	42.64 ± 8.23	37.83 ± 11.48
Blood differential × cells/mm^3^				
Total WBC	5,290.0 ± 1,327.0	8,340.0 ± 1,658.0	7,310.0 ± 2,083.0	4,913.0 ± 1,339.0
Macrophages	305.20 ± 70.10	837.90 ± 415.60	377.40 ± 211.30	245.80 ± 185.40
Lymphocytes	1,752.0 ± 397.30	2,685.0 ± 497.50	1,339.0 ± 303.30	^∗∗^764.90 ± 321.50
Neutrophils	3,055.0 ± 947.30	2,455.0 ± 613	4,665.0 ± 1,196	3,852.0 ± 842.90
Eosinophils	177.90 ± 53.55	^##^2,382.0 ± 706	^∗∗^377.40 ± 211.30	^∗∗^123.60 ± 51.07
BALF × 10^4^ cell/mL				
Total WBC	2.18 ± 0.71	^#^18.72 ± 7.30	^∗^^,##^7.30 ± 1.35	^∗^3.75 ± 0.76
Macrophages	1.92 ± 0.56	^#^9.44 ± 0.93	^$^3.38 ± 0.78	^∗^2.55 ± 0.43
Lymphocytes	0.20 ± 0.14	^##^1.19 ± 0.25	0.74 ± 0.41	^∗∗^0.20 ± 0.09
Neutrophils	0.05 ± 0.03	^##^0.95 ± 0.29	0.75 ± 0.23	0.61 ± 0.10
Eosinophils	0.01 ± 0.004	^#^7.12 ± 1.11	^∗^2.42 ± 0.89	^∗^0.39 ± 0.22
OVA-specific antibody (OD)				
IgE	0.07 ± 0.032	^##^1.23 ± 0.329	^∗∗^0.47 ± 0.055	^##^1.28 ± 0.45
IgG1	1.47 ± 0.317	2.08 ± 0.208	1.54 ± 0.321	2.45 ± 0.01
IgG2a	0.06 ± 0.008	^##^2.21 ± 0.405	^##^1.91 ± 0.473	1.17 ± 0.91
Organs/mice weight (%)				
Thymus	0.18 ± 0.01	^##^0.26 ± 0.02	^∗∗^0.15 ± 0.02	^∗^0.11 ± 0.01
Spleen	0.45 ± 0.04	^##^0.59 ± 0.01	^∗∗^0.49 ± 0.03	^∗^^,#,€^0.24 ± 0.01

**Table 2:** Metanuclear changes for cytotoxicity evaluation (pyknosis, karyorrhexis, and karyolysis) in mouse hepatocytes treated with sakuranetin. *p* > 0.05. Data represent mean ± SEM from 5 mice per group.

Groups	Metanuclear changes		
	Pyknosis	Karyorrhexis	Karyolysis
SAL	9 ± 6.7	22.5 ± 6.5	199 ± 8.5
OVA	16 + 2 ± 6.7	29.2 ± 13.5	196.5 ± 28.7
SK	17 ± 8.8	25.5 ± 9.3	211 ± 27

## Data Availability

The data used to support the findings of this study are available from the corresponding author upon request.

## References

[1] Possa S. S., Leick E. A., Prado C. M., Martins M. A., Tibério I. F. L. C. (2013). Eosinophilic inflammation in allergic asthma. *Frontiers in Pharmacology*.

[2] Bittencourt-Mernak M. I., Pinheiro N. M., Santana F. P. R. (2017). Prophylactic and therapeutic treatment with the flavonone sakuranetin ameliorates LPS-induced acute lung injury. *American Journal of Physiology-Lung Cellular and Molecular Physiology*.

[3] Santana F. P. R., Pinheiro N. M., Mernak M. I. B. (2016). Evidences of herbal medicine-derived natural products effects in inflammatory lung diseases. *Mediators of Inflammation*.

[4] Taguchi L., Pinheiro N. M., Olivo C. R. (2015). A flavanone from *Baccharis retusa* (Asteraceae) prevents elastase-induced emphysema in mice by regulating NF-*κ*B, oxidative stress and metalloproteinases. *Respiratory Research*.

[5] Sakoda C. P. P., de Toledo A. C., Perini A. (2016). Sakuranetin reverses vascular peribronchial and lung parenchyma remodeling in a murine model of chronic allergic pulmonary inflammation. *Acta Histochemica*.

[6] Toledo A., Sakoda C. P. P., Perini A. (2013). Flavonone treatment reverses airway inflammation and remodelling in an asthma murine model. *British Journal of Pharmacology*.

[7] Chen S.-M., Tsai Y. S., Lee S. W. (2014). *Astragalus membranaceus* modulates Th1/2 immune balance and activates PPAR*γ* in a murine asthma model. *Biochemistry and Cell Biology*.

[8] Chen J., Wang J.-B., Yu C.-H., Chen L.-Q., Xu P., Yu W.-Y. (2013). Total flavonoids of *Mosla scabra* leaves attenuates lipopolysaccharide-induced acute lung injury via down-regulation of inflammatory signaling in mice. *Journal of Ethnopharmacology*.

[9] de Oliveira J. F. F., Garreto D. V., da Silva M. C. P. (2013). Therapeutic potential of biodegradable microparticles containing *Punica granatum* L. (pomegranate) in murine model of asthma. *Inflammation Research*.

[10] Li J., Zhang B. (2013). Apigenin protects ovalbumin-induced asthma through the regulation of Th17 cells. *Fitoterapia*.

[11] Dong C., Davis R. J., Flavell R. A. (2002). MAP kinases in the immune response. *Annual Review of Immunology*.

[12] Meduri G. U., Kohler G., Headley S., Tolley E., Stentz F., Postlethwaite A. (1995). Inflammatory cytokines in the BAL of patients with ARDS. Persistent elevation over time predicts poor outcome. *Chest*.

[13] Chialda L., Zhang M., Brune K., Pahl A. (2005). Inhibitors of mitogen-activated protein kinases differentially regulate costimulated T cell cytokine production and mouse airway eosinophilia. *Respiratory Research*.

[14] Yin Y., Liu W., Dai Y. (2015). SOCS3 and its role in associated diseases. *Human Immunology*.

[15] Pu Q., Zhao Y., Sun Y. (2018). TRPC1 intensifies house dust mite–induced airway remodeling by facilitating epithelial-to-mesenchymal transition and STAT3/NF-*κ*B signaling. *The FASEB Journal*.

[16] Sun W., Xiao B., Jia A. (2018). MBD2-mediated Th17 differentiation in severe asthma is associated with impaired SOCS3 expression. *Experimental Cell Research*.

[17] Gavino A. C., Nahmod K., Bharadwaj U., Makedonas G., Tweardy D. J. (2016). STAT3 inhibition prevents lung inflammation, remodeling, and accumulation of Th2 and Th17 cells in a murine asthma model. *Allergy*.

[18] Zafra M. P., Cañas J., Mazzeo C. (2015). SOCS3 silencing attenuates eosinophil functions in asthma patients. *International Journal of Molecular Sciences*.

[19] Hasegawa T., Uga H., Mori A., Kurata H. (2017). Increased serum IL-17A and Th2 cytokine levels in patients with severe uncontrolled asthma. *European Cytokine Network*.

[20] GINA (2018). Global strategy for asthma management and prevention (2018 update). https://www.ginasthma.org.

[21] Harris T. J., Grosso J. F., Yen H. R. (2007). Cutting edge: an in vivo requirement for STAT3 signaling in TH17 development and TH17-dependent autoimmunity. *Journal of Immunology*.

[22] Murphy D. M., O’Byrne P. M. (2010). Recent advances in the pathophysiology of asthma. *Chest*.

[23] Kilkenny C., Browne W., Cuthill I. C., Emerson M., Altman D. G. (2010). Animal research: reporting *in vivo* experiments: the ARRIVE guidelines. *British Journal of Pharmacology*.

[24] Yoshizaki K., Brito J. M., Toledo A. C. (2010). Subchronic effects of nasally instilled diesel exhaust particulates on the nasal and airway epithelia in mice. *Inhalation Toxicology*.

[25] Weibel E. R. (1963). Principles and methods for the morphometric study of the lung and other organs. *Laboratory Investigation*.

[26] Brüggemann T. R., Fernandes P., de Mendonça Oliveira L., Sato M. N., de Arruda Martins M., Arantes-Costa F. M. (2017). Cigarette smoke increases CD8*α*^+^ dendritic cells in an ovalbumin-induced airway inflammation. *Frontiers in Immunology*.

[27] Caperuto L. C., Anhê G. F., Cambiaghi T. D. (2008). Modulation of bone morphogenetic protein-9 expression and processing by insulin, glucose, and glucocorticoids: possible candidate for hepatic insulin-sensitizing substance. *Endocrinology*.

[28] Pinheiro A. J. M. C. R., Gonçalves J. S., Dourado Á. W. A. (2018). *Punica granatum* L. leaf extract attenuates lung inflammation in mice with acute lung injury. *Journal of Immunology Research*.

[29] dos Santos Grecco S., Reimão J. Q., Tempone A. G. (2012). In vitro antileishmanial and antitrypanosomal activities of flavanones from *Baccharis retusa* DC. (Asteraceae). *Experimental Parasitology*.

[30] Kim K.-Y., Kang H. (2016). Sakuranetin inhibits inflammatory enzyme, cytokine, and costimulatory molecule expression in macrophages through modulation of JNK, p38, and STAT1. *Evidence-based Complementary and Alternative Medicine*.

[31] Yun J.-M., Im S. B., Roh M. K. (2014). *Prunus yedoensis* bark inhibits lipopolysaccharide-induced inflammatory cytokine synthesis by I*κ*B*α* degradation and MAPK activation in macrophages. *Journal of Medicinal Food*.

[32] Hernández V., Recio M. C., Máñez S., Giner R. M., Ríos J.-L. (2007). Effects of naturally occurring dihydroflavonols from *Inula viscosa* on inflammation and enzymes involved in the arachidonic acid metabolism. *Life Sciences*.

[33] Le Gros G., Ben-Sasson S. Z., Seder R., Finkelman F. D., Paul W. E. (1990). Generation of interleukin 4 (IL-4)-producing cells in vivo and in vitro: IL-2 and IL-4 are required for in vitro generation of IL-4-producing cells. *The Journal of Experimental Medicine*.

[34] Gavett S. H., O'Hearn D. J., Karp C. L. (1997). Interleukin-4 receptor blockade prevents airway responses induced by antigen challenge in mice. *The American Journal of Physiology*.

[35] Sjölander A., Baldwin T. M., Curtis J. M., Handman E. (1998). Induction of a Th1 immune response and simultaneous lack of activation of a Th2 response are required for generation of immunity to leishmaniasis. *The Journal of Immunology*.

[36] Jang T. Y., Jung A.-Y., Kwon S., Kim Y. H. (2018). Hypergravity enhances the therapeutic effect of dexamethasone in allergic asthma and rhinitis animal model. *PLoS One*.

[37] Ye W.-J., Xu W. G., Guo X. J. (2017). Differences in airway remodeling and airway inflammation among moderate-severe asthma clinical phenotypes. *Journal of Thoracic Disease*.

[38] Christianson C. A., Alam R. (2013). Mechanisms of sustained signalling in asthma. *Current Opinion in Allergy and Clinical Immunology*.

[39] Khorasanizadeh M., Eskian M., Gelfand E. W., Rezaei N. (2017). Mitogen-activated protein kinases as therapeutic targets for asthma. *Pharmacology & Therapeutics*.

[40] Huang L., Wang M., Yan Y. (2018). OX40L induces helper T cell differentiation during cell immunity of asthma through PI3K/AKT and P38 MAPK signaling pathway. *Journal of Translational Medicine*.

[41] Duan W., Chan J. H. P., Wong C. H., Leung B. P., Wong W. S. F. (2004). Anti-inflammatory effects of mitogen-activated protein kinase kinase inhibitor U0126 in an asthma mouse model. *Journal of Immunology*.

[42] Kujime K., Hashimoto S., Gon Y., Shimizu K., Horie T. (2000). p38 mitogen-activated protein kinase and c-Jun-NH2-terminal kinase regulate RANTES production by influenza virus-infected human bronchial epithelial cells. *Journal of Immunology*.

[43] Seki Y., Inoue H., Nagata N. (2003). SOCS-3 regulates onset and maintenance of TH2-mediated allergic responses. *Nature Medicine*.

[44] Tas S. W., Maracle C. X., Balogh E., Szekanecz Z. (2016). Targeting of proangiogenic signalling pathways in chronic inflammation. *Nature Reviews Rheumatology*.

[45] Martínez-Rivera C., Crespo A., Pinedo-Sierra C. (2018). Mucus hypersecretion in asthma is associated with rhinosinusitis, polyps and exacerbations. *Respiratory Medicine*.

[46] Daviskas E., Anderson S. D. (2006). Hyperosmolar agents and clearance of mucus in the diseased airway. *Journal of Aerosol Medicine*.

[47] Atherton H. C., Jones G., Danahay H. (2003). IL-13-induced changes in the goblet cell density of human bronchial epithelial cell cultures: MAP kinase and phosphatidylinositol 3-kinase regulation. *American Journal of Physiology Lung Cellular and Molecular Physiology*.

[48] Moon Y. J., Wang X., Morris M. E. (2006). Dietary flavonoids: effects on xenobiotic and carcinogen metabolism. *Toxicology In Vitro*.

[49] Dahan A., Altman H. (2004). Food-drug interaction: grapefruit juice augments drug bioavailability—mechanism, extent and relevance. *European Journal of Clinical Nutrition*.

